# Development of a bleeding scale and hemostasis algorithm in cranial neurosurgery

**DOI:** 10.1016/j.heliyon.2023.e22806

**Published:** 2023-11-29

**Authors:** Ilker Y. Eyüpoglu, Jochen Tuettenberg, Karl-Michael Schebesch, Ralf Buhl, Jürgen A. Hampl, George D. Kiriyanthan, Christian Scheiwe

**Affiliations:** a Department of Neurosurgery, University of Erlangen-Nuremberg, Germany; b Department of Neurosurgery, SHG Klinikum Idar-Oberstein GmbH, Germany; c Department of Neurosurgery, Medical Center of the University of Regensburg, Germany; d Department of Neurosurgery, Städtisches Klinikum Solingen, Germany; e Center of Neurosurgery, Department of General Neurosurgery, University of Cologne, Faculty of Medicine and University Hospital Cologne, Germany; f Department of Neurosurgery, Städtisches Klinikum Karlsruhe, Germany; g Department of Neurosurgery, Medical Center - University of Freiburg, Germany

**Keywords:** Hemostasis, Intracranial bleeding, Bleeding scale, Hemostasis algorithm, Bleeding score, Topical hemostats

## Abstract

Effective hemostasis is crucial in neurosurgery as anatomical and functional considerations reduce tolerance for any bleeding. The classification of bleeding severities is a necessary step to enable neurosurgeons to counteract bleeding during surgery.

Even though bleeding scales are used for a variety of surgical specialties, they cannot be transferred to cranial neurosurgery without adaption, and no consensus on the nature of such a classification exists to date. Moreover, there is plethora of topical hemostasis products with diverse mechanisms of action and application available. Clinical studies investigating those products used in neurosurgery did not define standardized procedures. This article demonstrates the systematic establishment of both a bleeding scale and a hemostasis algorithm to close this gap in the assessment of intracranial bleeding.

The expert panel consisting of 7 members from different neurosurgical centers developed a qualitative bleeding scale with the peculiarities of neurosurgical procedures, based on the experience of each member in daily practice. The hemostasis algorithm is a recommendation for neurosurgeons to aid in the decision-making process to control any sort of bleeding, taking into account the rational use of available hemostatics, depending on type and location of bleeding, as well as the mechanism of action of such agents. Effectiveness of hemostasis, surgery times and economic costs can be optimized by applying the algorithm in daily practice.

## Abbreviation list

ASA: American Society of Anesthesiologists
CE: European Conformity
EMA: European Medicines Agency
FDA: Food and Drug Administration
NOACS: New oral anticoagulants
VIBe scale: Validated Intraoperative Bleeding Scale

## 1 Introduction

Successful and effective hemostasis is crucial in surgical procedures. Insufficient hemostasis increases the risk of perioperative morbidity and mortality.

Bleeding severity scales validated in surgical procedures help to avoid risks as the use of inappropriate hemostatic methods or agents. These standardized criteria also allow comparison of postoperative outcome and the effectiveness of hemostatic methods and topical hemostats in clinical studies [1].

A bleeding scale specific to intracranial operations has not been established despite this being of high importance. The need of a neurosurgery-specific bleeding scale bases on the peculiarities specific to the central nervous system which need a tailored approach to hemostasis in order to prevent primary and secondary neuronal damage.

An existing bleeding scale was taken as the foundation and compared to the daily practice of the expert consortium [2]. The first step was to develop the cranial neurosurgery-specific bleeding scale and in a second step consequently establish an algorithm as guideline of possible treatment methods and products in relation to the severity and localization of cranial bleeding.

The use of hemostatic agents can be an indispensable intraoperative feature across all surgical specialties, where the range of applied products and methods corresponds to the scope of the intervention concerned [[3], [4], [5], [6]].

Clinical studies investigating topical hemostatic agents have not been characterized by standardized definitions or classifications for intraoperative bleeding severity or hemostasis [7,8].

As a result, the labeling claims of hemostatic agents unfortunately remain ambiguous. This generic manner of labeling hinders the surgeons' selection of the appropriate agent by inadvertently concealing possible meaningful clinical differences of the respective agents. Furthermore, a disproportionate increase in healthcare costs and waste of medical resources might be the consequences [[9], [10], [11], [12], [13], [14]]. Whereas the listed aspects can be improved by paying thorough attention to hemostasis, independent risk factors are the use of antiplatelet and anticoagulation agents - often obligatory due to underlying comorbidities [1,15,16].

This requires a systematic classification of the currently very generic labelling of available hemostatic agents. The differentiation of hemostatic products into passive and active agents was taken into consideration [17].

The rationale for the development of a hemostasis algorithm based on a qualitative bleeding scale specific to cranial neurosurgery is presented in this paper. We aimed to establish a reliable and robust bleeding scale specific to neurosurgery allowing a quick and uncomplicated categorization and assess of any intraoperative bleeding situation. The hemostatic algorithm should help neurosurgeons make a decision about how to treat the bleeding and which type of hemostatic agent is appropriate. Furthermore, this consensus-based approach can act as a standard for clinical studies with hemostatic agents.

## 2 Material and methods

The expert panel consisted of neurosurgical specialists from major German neurosurgical centers with at least 20 years of experience consulting in this discipline, according to the list of authors. All members have various professional neurosurgical training backgrounds and work in leading positions in university or tertiary care hospitals. They therefore represent a broad distribution of different schools of neurosurgery, enriched by different stays abroad of the individual participants and their mentors.

Over the course of several advisory board meetings from 2017 to 2019, the consortium established a consensus of need and developed the hemostasis algorithm based on the bleeding scale specific to cranial neurosurgery. Several examples of intracranial bleeding situations were presented and classified in several open discussion rounds until consensus was reached. Therefore, this was not a Delphi consensus as it were open discussions and the validation process of the bleeding scale took place during the meetings. The developed bleeding scale allows a qualitative assessment of different bleeding situations in a first step with application of different measures to reach hemostasis according to the hemostasis algorithm in a second step.

The multi-factorial algorithm was developed in the same manner. Each step of a cranial neurosurgical operation was regarded separately discussing the different used methods to reach hemostasis in daily practice. Integrated into the algorithm were:

i) Severity grades (Bleeding scale, Table 1)

**Table 1:** Neurosurgical bleeding scale according to expert consortium recommendation and daily practice.

BLEEDING SCALE	
**Grade 0**	no bleeding
**Grade I**	bleeding stoppable with simple physical means
**Grade II**	**a** non eloquent, elaborate hemostasis: time-consuming or additive means
	**b** eloquent, elaborate hemostasis: time-consuming or additive means
**Grade III**	local bleeding, immediate hemostasis requiring additive means
**Grade IV**	diffuse bleeding, immediate hemostasis requiring additive means
ii) Localization of the cranial bleeding: sinus, dura, parenchyma, tumor, bone, and soft tissue (see Table 2).

**Table 2:** Hemostasis algorithm in cranial neurosurgery. The grading in the first column corresponds to the bleeding scale in Table 1.

	SINUS	DURA	PARENCHYMA	TUMOR	BONE	SOFT TISSUES
GRADE I	1. Head elevation 2. Bipolar coagulation 3. Cellulose, collagen, cotton, gelatin	1. Bipolar coagulation 2. Tenting suture 3. Cellulose, collagen, cotton, gelatin	1. Saline irrigation 2. Cellulose, cotton 3. Bipolar coagulation 4. Collagen, gelatin, polysaccharide powder (hydrogen peroxide)	1. Bipolar coagulation (cellulose, cotton, saline irrigation)	1. Wax	1. Bipolar coagulation 2. Compression
**GRADE** **II**	1. Head elevation 2. Bipolar coagulation 3. Cellulose, collagen, cotton, gelatin 4. Coated patch, fibrin, muscle or galea patch, sealant, suture	1. Bipolar coagulation 2. Tenting suture 3. Cellulose, collagen, cotton, gelatin	1. **a** Saline irrigation 2. Bipolar coagulation 3. Cellulose, cotton 4. Collagen, gelatin, polysaccharide powder (hydrogen peroxide) 1. **b** Saline irrigation 2. Cellulose, cotton 3. Bipolar coagulation 4. Collagen, gelatin, polysaccharide powder (hydrogen peroxide) + coated patch, flowable	1. Bipolar coagulation 2. Cellulose, cotton 3. Saline irrigation 4. Coated patch, flowable	1. Wax 2. Diamond burr	1. Bipolar coagulation 2. Compression (hydrogen peroxide)
**GRADE** **III**	1. Coated patch, (hemo)clip, suture		1. Bipolar coagulation 2. Coated patch, flowable, (hemo)clip	1. Bipolar coagulation 2. Coated patch, (hemo)clip	1. Wax 2. Diamond burr	1. Bipolar coagulation 2. Compression 3. (hemo)clip, ligation
GRADE IV			1. Optimization of systemic coagulation 2. Cotton 3. Coated patch, flowable	1. Optimization of systemic coagulation 2. Cotton 3. Coated patch, flowable		1. Optimization of systemic coagulation 2. Compression
iii) Available passive and active hemostatic agents: gelatins, collagen, oxidized cellulose, fibrin sealants, thrombin/flowables, and coated patches (Table 4),

**Table 3:** Evidence levels of literature specific to neurosurgery and hemostatic product classes.

	Class Ia	Class Ib	Class IIa	Class IIb	Class III	other
GELATIN/OTHER		1			6	
COLLAGEN			2			
OXIDIZED CELLULOSE			4			23
THROMBIN/FLOWABLES			1		6	
FIBRIN SEALANT						3
COATED PATCHES			2	1

**Table 4:**
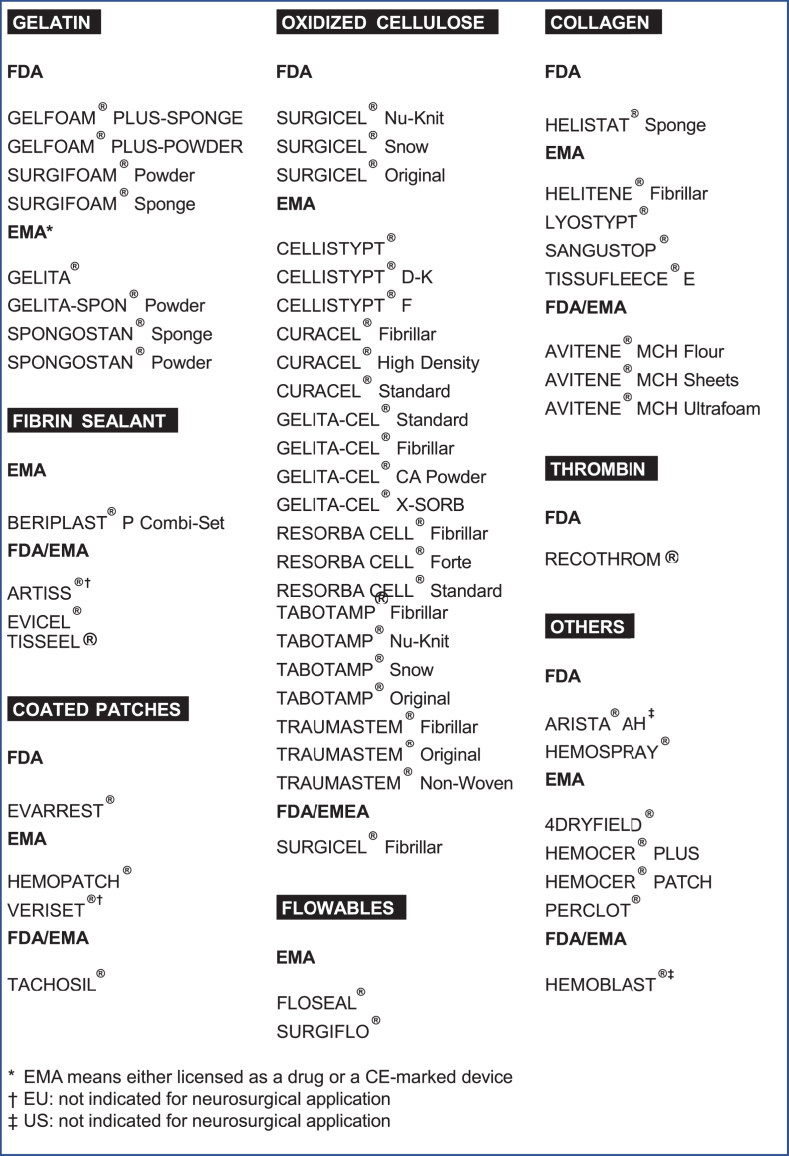
Hemostatic products currently available.

A systematic review of the literature for the different hemostatic agents in clinical research and experience was performed in PubMed and Cochrane. Search terms covered the available hemostatic agents combined with “hemostasis” and “neurosurgery”. In cases with no results or publications with evidence level < III, the terms were adapted to “hemostatic agent/product” plus “hemostasis” and “surgery”. The search timeframe was open start until March 31st, 2019. The search results were classified according to their evidence levels for clinical studies as suggested by the US Agency for Healthcare Research and Quality (see Table 3 for literature search results).

The products searched, discussed, and listed in this article are all authorized by the Food and Drug Administration (FDA) and/or European Medicines Agency (EMA) or European Conformity (CE) - marked for application in surgery. Listed are the most commonly used hemostatic products with no claim for completeness (Table 4).

## 3 Results

### 3.1 Development of the cranial bleeding scale

In the past some bleeding scales (i.e. Validated Intraoperative Bleeding (VIBe) Scale [2]) were mostly not tested and evaluated in clinical trials but pre-clinically. Some of the existing scores are quantitative but specific to a certain specialty or anatomical region. Besides, some provided agents exclude application in neurosurgery [[2], [17], [18], [19]]. The bleeding scale presented in this article bases on daily practice in cranial neurosurgery. It was not validated in animal models or in vitro. However it is common practice to establish classifications by statistical analysis or literature research, as also seen with the American Society of Anesthesiologists (ASA) criteria.

The bleeding severity was classified into grades 0-IV with increasing severity and necessity to act quickly and efficiently (see Table 1). Grade I bleeding was defined as mild to moderate (estimated rate of blood loss <10 ml/min). Grade II bleeding does not necessarily have a higher bleeding rate, but requires more elaborate hemostasis. If the amount of blood is low, this can also be done in the further course of the operation, but mandatory before closing the dura. According to functional considerations, Grade II was divided into IIa and IIb to differentiate between eloquent and non-eloquent areas. In eloquent brain areas, certain hemostatic measures such as bipolar coagulation can only be used to a limited extent, otherwise irreversible neurological deficits threaten. A severe local bleeding (estimated rate of blood loss <50 ml/min), which makes further surgery without immediate hemostasis impossible (usually arterial bleeding, e.g. in case of aneurysm rupture) was classified as grade III whereas severe diffuse bleeding (e.g. highly vascularized large tumor) with need for immediate hemostatic measures was classified as grade IV. , since on the one hand the lack of overview makes it impossible to continue operating and on the other hand relevant blood loss occurs in a short time. In contrast, for example in the case of a grade 2b bleeding, surgery may continue and the bleeding can also be controlled first by tamponing, since there is a functional, but no vital threat from the bleeding.

### 3.2 Development of the cranial hemostasis algorithm

The current spectrum of hemostatic methods and means consists of primary physical methods, such as head elevation, blood pressure control, the use of bipolar forceps, irrigation with or without hydrostatic elements, hydrogen peroxide saturated cottonoids. Secondary methods include passive hemostatic products like cellulose mesh or gelatin sponges [[20], [21], [22], [23], [24], [25], [26], [27], [28]]. Tertiary methods make use of active hemostats like fibrin sealant, flowables and advanced patches. These patches are separated into first-generation alloplastic material like fibrinogen/thrombin coated fleece and second-generation hemostatic compounds like polyethylene glycol-coated biomaterial [[29], [30], [31], [32], [33], [34]].

The application of the available hemostatic products on the market depends on a number of factors, i.e., the patient's coagulation status or the type of bleeding. Thus, the different kinds of bleeding require an appropriate approach. As presented in Table 2, the localization of bleeding was divided into sinus, dura, parenchyma, tumor, bone, and soft tissue - each necessitating separate evaluation and treatment.

Bleeding severity according to the abovementioned bleeding scale was implemented.

Table 2 presents the matrix of bleeding grades and localizations where a neurosurgeon can find the appropriate method of hemostasis. Different options are provided in each case to accommodate the local and personal preferences of each institution and/or surgeon.

The algorithm also includes methods of daily practice (e.g., bone wax, coagulation, or sutures).

### 3.3 Overview and classification of hemostatic agents

**Fig. 1:**
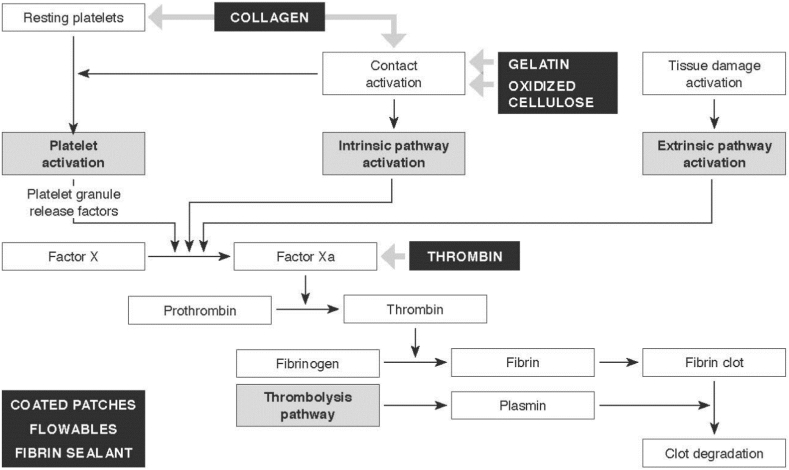
Shows the position of the product groups within the coagulation cascade.

A necessary step is to recognize how different hemostatic products interfere in the coagulation cascade (see Fig. 1, generic product groups are marked in black).

The hemostatic product groups engage differently within the coagulation cascade by either activating the platelets and therefore the primary hemostasis process, or directly influencing the coagulation cascade and therefore stimulating secondary hemostasis (as shown in Fig. 1). Thus, they qualify for different bleeding types and severity grades (see Table 2).

Part of the work of the expert consortium was a systematic literature review for clinical evidence of those hemostatic agents to underline their own experience in managing cranial bleeding (results see Table 3). There is only a limited number of articles describing the different hemostatic agents listed above. The number of clinical studies is even lower, emphasizing the necessity for an objective way to classify the severity of bleeding and thus the efficacy of hemostasis achieved with these products.

In addition to the literature research regarding the different local hemostatic agents, the expert consortium conducted a search for available products on the market with respect to FDA and EMA or European Conformity (CE)-marked approval with no claim for completeness.

Product classes include passive products, such as oxidized celluloses, collagens, gelatin as well as active products like fibrin sealants, thrombin/flowables, coated patches and others, mostly hemostatic powders, as also seen in Table 4. Some of these listed products are not yet approved by the FDA or EMA or are CE-marked for neurosurgical applications.

Passive hemostatic agents, which only support platelet activation, are natural compounds, mostly absorbable with a porous surface to facilitate quick platelet adherence.

Active hemostatic agents interfere in some way with the coagulation cascade. Fibrin sealants consisting of fibrinogen and thrombin directly form a fibrin network matrix in which erythrocytes and platelets adhere to form a stable fibrin clot. Similar to these fibrin sealants, flowables interact directly with the coagulation cascade, providing the thrombin to form the fibrin clot.

Moreover, advanced coated patches are combination products, usually of a collagen base, with either thrombin/fibrinogen or polyethylene glycol coating. They rely on a combination of physical and biological/chemical reactions, whereby the matrix of the patch, together with the respective component-coating, supports hemostasis independent of the patient's own coagulation.

The last group “other products” i.e., hemostatic powders, like powdered polysaccharides, which can bind the water in the blood, leading to a concentration of the platelets and coagulation factors in the blood to support hemostasis.

## 4 Discussion

Hemostasis of cranial bleeding differs substantially compared to other surgical specialties. There is a general lack of reliable classification systems that can be applied in cranial neurosurgery [[18], [19], [20]]. No objective recommendations for daily practice exist, leading to a highly subjective, sometimes cost-driven decision in the choice of hemostatic agents/methods. Additionally, the subjective approach undermines the opportunity to evaluate and compare different hemostatic methods in a reliable and statistically valuable clinical study.

The aim of the presented bleeding scale and hemostasis algorithm was to categorize cranial bleeding according to localization and bleeding severity in a qualitative way. Recommendations which methods and products are most suitable for hemostasis in the respective cases are provided. A measure for the clinical comparison of different methods is also proposed. This presents new respected means for labelling claims of hemostatic agents. Standardized criteria can warrant patients not being subjected to avoidable risks (failed, delayed, or inappropriate treatment).

In cardiac surgery, several attempts have failed to broadly implement specific and detailed bleeding scales, such as the E-CABG (additive score for classification of severity of intraoperative and postoperative bleeding after adult cardiac surgery) or the Universal Definition for Perioperative Bleeding (UDPB) score [35,36].

In general surgery, few bleeding scores, such as the Spot Grade and the Validated Intraoperative Bleeding (VIBe)-Scale, are known for different surgical specialties [ [2,18]]. The VIBe-Scale consists of a simple and valid 5-score construct to describe bleeding intensities in a qualitative and quantitative manner as well as by the anatomical appearance of the bleeding origin. Up to this day, the VIBe-Scale is the first FDA-accepted bleeding scale and is used for hemostatic product registration. Nevertheless, the use and clinical implementation of these bleeding scales has hitherto been rather low.

The intracranial bleeding scale is based on the VIBe-Scale as an example of an existing bleeding scale in surgery and was developed taking into account the knowledge of the expert panel in daily practice. There was no quantitative assessment of the blood loss over time but the simple structure of the scale with only four different levels allows a reproducible categorization of the bleeding situation and based on this the surgeons decision in which way to react [2]. The main objective of the cranial hemostasis algorithm is to act as a guide for daily practice by neurosurgeons in cases of cranial bleeding in order to facilitate an objective outcome, time-, and cost-efficient approach for successful hemostasis. The step-by-step approach includes a variety of questions to be answered, such as the safety of the chosen method, advantages, and disadvantages of each method/product. Time and costs are also taken into account. The appropriate product will be chosen depending on efficacy and based on experience. This is where the hemostasis algorithm based on the new bleeding scale helps to close a possible experience gap and reduces the possibility for unnecessary discussions. There are well standardized applications for surgery for hemostatic products which might already help, but they cannot equally be applied to all cases of cranial bleeding. The hemostasis algorithm presented here is therefore a hands-on approach in these special cases.

The expert panel took all these questions and challenges into account when trying to classify the bleeding grades according to localization, and assigning the products and methods for hemostasis. Based on the new algorithm, a neurosurgeon can now decide quickly and efficiently which kind of hemostatic agent/method may be used.

The expert panel consists solely from neurological centers of a single country. We think nevertheless, that due to the fact of different training backgrounds in each neurosurgical department together with international consultants and abroad fellowships of board members as well as their mentors, this expert panel represents a pretty wide and international range of different opinions on how to deal with intracranial bleeding situations. Of course, there may be other preferred methods of hemostasis in individual countries, but these surgeons can also profit from the presented hemostasis algorithm as they may find a new point of view which method is considered adequate by other expert neurosurgeons in different bleeding situations.

A systematic differentiation of hemostatic products into passive (or mechanical) and active agent can help in choosing the right product for a specific intensity of bleeding. While passive hemostatic products only provide a physical structure for platelet activation, their mechanism of action fully relies on the patient's ability to generate clotting factors. Passive hemostats may not be effective in patients with an impaired coagulation status or across all bleeding types. In contrast, active hemostats either participate at the end of the coagulation cascade and bypass the initial steps of the clotting cascade or actively seal the tissue surface to promote hemostasis. Active hemostats are effective regardless of the patient's coagulation and across a broader range of bleeding grades [17].

As presented in Table 2, the hemostasis algorithm suggests a use of mechanical or passive agents for lower grade bleeding in most of the localizations, whereas active agents are mainly advised for bleeding of higher severity.

Grade 4 bleeding (only for parenchyma, tumor, or soft tissue) require, besides the preoperative optimization of systemic coagulation, further steps additional to the hemostatic agents presented and discussed here: an optimization of systemic coagulation and application of further treatment options.

In some cases, antidotes exist (e.g., Prothrombin Complex Concentrate (PCC) for Vitamin K antagonist, Desmopressin for Acetyl Salicylic Acid), but new oral anticoagulants (NOACS) are extremely difficult in patients with impaired coagulation status and should be advised preoperatively (higher risk, complications). Children can be very problematical as well. Transfusion and administration of platelets may be necessary. If possible, neurosurgical patients with impaired coagulation status should be referred to specialized centers. These factors all have to be taken into consideration for the assessment of cranial bleeding and the subsequent choice of hemostatic agents/methods.

The hemostasis algorithm also presents an additional opportunity as a learning tool for inexperienced neurosurgeons to close the experience gap and augment an objective training setup for upcoming colleagues. The order of actions recommended in the hemostasis algorithm is the order the expert panel would suggest for applying the listed products and methods, but needless to say it can be adapted to personal taste or institution practice of each neurosurgeon and team.

It is also worth putting the economic aspects into context. On the one hand, there might be no need to use advanced products. On the other hand, in the instance of more severe cranial bleeding, wasting ineffective products is also inefficient as opposed to choosing the most effective hemostatic agents right away. A further benefit are the possible shorter operation times by implementing the tailored method for hemostasis when set against the costs of the applied hemostatic agents, ensuring that the appropriate product is used promptly for the appropriate type of bleeding.

It is important to mention here that the hemostatic algorithm will not be completely applicable all over the world since the factor of time might be more important in some countries where resources are more limited. Some products might not even be available at all.

Notwithstanding, the bleeding scale and hemostasis algorithm presented here represent a new and overdue standardization of the application of hemostatic agents/methods in neurosurgery, specifically in instances of cranial bleeding.

As mentioned before, due to the low evidence level of the literature concerning hemostatic agents, especially in the field of neurosurgery, there is a pressing need for further studies to confirm the findings and recommendations. Clinical studies in this context, including observational studies, provide the opportunity to validate the expert panel's work and deliver better and more objective recommendations to neurosurgeons in cases of cranial bleeding.

## 5 Limitations

There are some limitations in the context of the algorithm presented here. The bleeding scale is not validated besides within the expert panel and does not assess the amount of blood loss over time, but solely relies on daily practice and expert opinions. The level of evidence in the literature of the classified hemostatic agent groups is rather low. Additionally, the hemostasis algorithm might not work for all patient groups, so it may be necessary to make exceptions (e.g., children, allergic patients, Jehovah's Witnesses). Overall validity could be improved by the daily use and experience of surgeons and additional observational studies.

## Funding

All authors report grants from Baxter Deutschland GmbH.

## Additional information

No additional information is available for this paper.

## CRediT authorship contribution statement

**Ilker Y. Eyüpoglu:** Conceptualization, Data curation, Investigation, Methodology, Supervision, Validation, Writing - original draft. **Jochen Tuettenberg:** Conceptualization, Data curation, Formal analysis, Methodology, Supervision, Writing - review & editing. **Karl-Michael Schebesch:** Conceptualization, Data curation, Formal analysis, Investigation, Supervision, Validation, Writing - review & editing. **Ralf Buhl:** Conceptualization, Data curation, Formal analysis, Investigation, Methodology, Validation, Writing - review & editing. **Jürgen A. Hampl:** Conceptualization, Data curation, Formal analysis, Investigation, Methodology, Supervision, Validation, Writing - review & editing. **George D. Kiriyanthan:** Conceptualization, Data curation, Formal analysis, Investigation, Methodology, Supervision, Validation, Writing - review & editing. **Christian Scheiwe:** Conceptualization, Data curation, Formal analysis, Investigation, Methodology, Validation, Visualization, Writing - original draft, Writing - review & editing.

## Declaration of competing interest

The authors declare the following financial interests/personal relationships which may be considered as potential competing interests:This work was supported by Baxter Deutschland GmbH, Edisonstr. 4 85,716 Unterschleissheim, Germany. All authors received financial support during advisory board meetings.
